# Cryo-electron structures of the extreme thermostable enzymes Sulfur Oxygenase Reductase and Lumazine Synthase

**DOI:** 10.1371/journal.pone.0275487

**Published:** 2022-10-03

**Authors:** Mohamed A. Sobhy, Lingyun Zhao, Dalaver Anjum, Ali Behzad, Masateru Takahashi, Muhammad Tehseen, Alfredo De Biasio, Rachid Sougrat, Samir Hamdan

**Affiliations:** 1 Bioscience Program, Division of Biological and Environmental Sciences and Engineering, King Abdullah University of Science and Technology, Thuwal, Saudi Arabia; 2 Electron Microscopy Core Labs, King Abdullah University of Science and Technology, Thuwal, Saudi Arabia; University of Queensland, AUSTRALIA

## Abstract

Thermostable enzymes have the potential for use in a wide variety of biotechnological applications. Cryo-electron microscopy (cryo-EM) enables the imaging of biomolecules in their native aqueous environment. Here, we present high resolution cryo-EM structures of two thermostable enzymes that exhibit multimeric cage-like structures arranged into two different point-group symmetries. First, we determined the structure of the Sulfur Oxygenase Reductase (SOR) enzyme that catalyzes both the oxygenation and disproportionation of elemental sulfur in Archea and is composed of 24 homomeric units each of MW ≃ 35 kDa arranged in octahedral symmetry. The structure of SOR from *Acidianus ambivalens* (7X9W) was determined at 2.78 Å resolution. The active site of each subunit inside the central nanocompartment is composed of Fe^3+^ coordinated to two water molecules and the three amino acids (H86, H90 and E114). Second, we determined the structure of Lumazine Synthase (LS) from *Aquifex aeolicus* (7X7M) at 2.33 Å resolution. LS forms a cage-like structure consisting of 60 identical subunits each of MW ≃ 15 kDa arranged in a strict icosahedral symmetry. The LS subunits are interconnected by ion-pair network. Due to their thermostability and relatively easy purification scheme, both SOR and LS can serve as a model for the catalytic and structural characterization of biocatalysts as well as a benchmark for cryo-EM sample preparation, optimization of the acquisition parameters and 3D reconstruction.

## Introduction

Multimeric cage proteins exist in many life forms and are involved in various metabolic processes [1–3]. Several studies attempted to explain how the individual subunits self-assemble to form large protein biostructures [4, 5]. Cage proteins possess three types of surfaces; an inner surface that faces the central cavity, an outer surface that faces the external environment, and inter‐subunit surfaces [6, 7]. Similar to other enzymes, multimeric cage proteins catalyze biochemical reactions [8–10] in addition to having unique features such as the extraordinary thermal stability of proteins from hyperthermophiles [11, 12] and ion storage properties [13]. Recently, there has been increasing interest in the self-assembly of proteins for the construction of nanocompartments and encapsulation of bioactive products for drug delivery [14].

The metabolism of elemental sulfur and reduced inorganic sulfur compounds provide the energy for the growth of many microorganisms [15]. Also, these reactions are involved in technological applications such as biomining of base and precious metal sulfide ores [16, 17]. Chemolithoautotrophic microorganisms use sulfur as energy source in light-independent ecosystems such as hot springs and hydrothermal vents [18–20]. In these microorganisms, the enzyme Sulfur Oxygenase Reductase (SOR) catalyzes the sulfur disproportionation reaction in an oxygen-dependent manner releasing sulfite, thiosulfate, and hydrogen sulfide as products [21]. SOR was first purified from the thermophilic, chemolithotrophic sulfur-metabolizing archaea *Acidianus* species [22, 23]. Besides thermoacidophilic archaea, SOR homologs were also discovered in bacteria [24, 25]. These microorganisms are capable of producing energy through the oxidization of elemental sulfur to sulfate and simultaneously reducing sulfur to sulfide in the absence of molecular hydrogen [26]. SOR is a homomultimeric thermostable enzyme operating in the temperature range 50°C to 108°C with maximum activity at 85°C [21, 23]. Under aerobic conditions, SOR can oxidize and reduce elemental sulfur simultaneously releasing sulfite, thiosulfate and hydrogen sulfide, where thiosulfate is produced as a product of the chemical reaction between sulfite and sulfur (Eq 1) [23, 27]. In this work, SOR refers to the sulfur-oxidizing enzyme from *Acidianus ambivalens* which grows optimally at 80°C and pH 1–3 and is studied as a model organism for sulfur metabolism [25, 28, 29].

SOR is composed of twenty-four monomeric units each ≃ 35 kDa arranged in octahedral symmetry with a total molecular weight of 871 kDa [21]. SOR has analogous architecture to that of ferritins which are relatively smaller iron storage proteins with an inner cavity and have the same point-symmetry group [21, 30–32]. The outer surface of SOR has six characteristic nanopore protrusions constituted by four-helix bundles that belong to four individual monomers at the four-fold pseudo-symmetry axes surrounded by ring-shaped grooves [32]. These structures form hydrophobic, apolar inner surface channels on the enzyme surface and give access to linear sulfur compounds into a central positively charged nanocompartment [21, 33]. The active site in each monomer is composed of non-heme iron site ligated by three amino acids and is thought to catalyze both the oxidation and reduction of sulfur [32].

Another example of a multimeric protein with exceptional thermostability is Lumazine Synthase (LS) which has a different symmetry group and distinct number of subunits. LS is involved in the biosynthesis of riboflavin (vitamin B_2_) [34]. Riboflavin is produced in plants, bacteria and yeast, whereas animals rely on exogenous sources of this vitamin [35]. Riboflavin-derived coenzymes are essential for all cellular organisms. Compounds interfering with the biosynthesis of folic acid, a vitamin that shares structural and biosynthetic similarities with riboflavin, have been successfully used as chemotherapeutic agents [36–39]. LS catalyzes the formation of 6,7-dimethyl-8-ribityllumazine by condensation of 5-amino-6-(D-ribitylamino)uracil with 3,4-dihydroxy-2-butanone 4-phosphate. LSs exist in different organisms such as *Saccharomyces cerevisiae* [40], spinach [41] and *Bacillus subtilis* [9, 42, 43], and possess similar structures [21, 31, 32]. LS from the hyperthermophilic bacterium *Aquifex aeolicus* living in hot springs of up to 90°C is extremely thermostable with a 119.9°C melting temperature, which is ~27°C higher than that of the next thermostable LS from *B*. *subtilis* [44]. LS from *Aquifex aeolicus* has the largest number of ion-pairs per subunit, where the charged residues represent the largest accessible surface while the hydrophobic residues represent the smallest surface. Its exceptional thermostability is attributed to the role of ion-pair networks in linking the adjacent subunits and optimization of hydrophobic and ionic contacts [44–46].

Over the past years, improvements in transmission electron microscopes, detectors and image processing software have empowered the exponential growth in the determination of high-resolution structures of macromolecular complexes by cryo-electron microscopy (cryo-EM) [47, 48]. The highest reported resolution is for apoferritin determined at 1.25 Å resolution using a monochromator and a spherical aberration corrector built into a transmission electron microscope with a direct electron detector [49]. This surpassed the 1.54 Å resolution structure of apoferritin acquired using a microscope equipped with a cold field emission gun electron source and an energy filter [50]. Also, 1.8 Å resolution structure of β-galactosidase has been reported [51]. On the other hand, several software packages have been developed for single-particle cryo-EM data processing including RELION [52, 53], EMAN2 [54], Cryosparc [55], and SPHIRE [56]. Live preprocessing capabilities during cryo-EM data acquisition have been incorporated in some software packages such as Cryosparc and RELION. Other packages such as Warp [57] and Scipion [58] implement a workflow-based approach to integrate the capabilities from different software packages. The continuous development in the computing power and the implementation of graphical processing unit (GPU) acceleration for image processing in cryo-EM software packages have enabled major improvements in data processing and 3D reconstructions [59].

In this study, we present the cryo-EM structures of two thermostable multimeric enzymes that have different point-symmetry groups; namely SOR from *Acidianus ambivalens* and LS from *Aquifex aeolicus*. The reported cryo-EM structures of the two enzymes including their active sites correlate very well with the x-ray crystal structures [21, 44]. The motivations for this work are: first, studying the structural features of thermostable enzymes by cryo-EM in near native conditions without the need for crystallization. Second, bring to the readers’ attention thermostable enzymes as potential candidates for benchmarking cryo-EM. The extreme thermostability of these enzymes renders their structures very stable and makes them easier to purify. Therefore, we propose that the field thermostable enzymes can benefit from the advancements in cryo-EM imaging and in return the stability of these enzymes can contribute to obtaining high resolution maps for benching cryo-EM.

## Results

### Cryo-EM structure of Sulfur Oxygenase Reductase (SOR)

**Fig 1:**
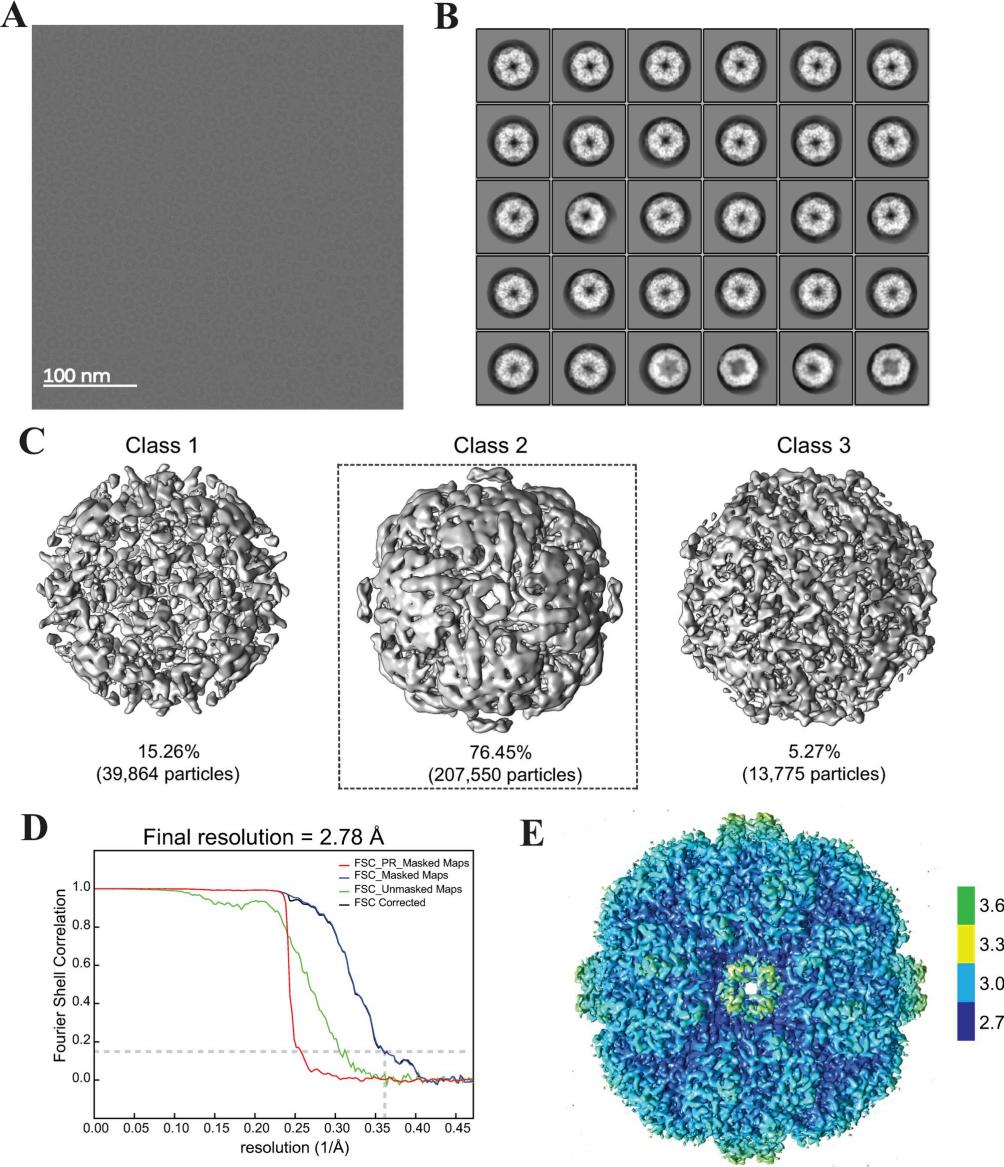
Cryo-EM imaging and 3D reconstruction of Sulfur Oxygenase Reductase (SOR) cryo-EM map. (**A**) Representative micrograph of SOR enzyme acquired in super-resolution mode at 130 kx magnification. The scale bar represents 100 nm. (**B**) The selected 2D classes contained 261,189 particles. All the shown 2D classes were subsequently used for 3D classification. **(C)** The 3D Class 2 reproduced the correct protein geometry with six-channel-like protrusions on its outer surface and constituted the major population of the particles (76.46%). The other two 3D classes (1 and 3) contained incomplete particles and were excluded from the refinement. (**D**) The Fourier shell correlation plot (FSC) shows the resolution of unmasked and masked maps. At FSC = 0.143, the final resolution of the cryo-EM map is 2.78 Å. (**E**) The final cryo-EM map is color-coded with the local resolution estimate obtained from RELION. The data processing pipeline flowchart for SOR is depicted in **S2 Fig**.

Recombinant SOR was expressed in *Escherichia coli* and vitrified for cryo-EM analysis as described in the Materials and Methods section. The size-exclusion chromatogram and SDS-PAGE gel image for the SOR sample are shown in **S1 Fig**. The acquisition parameters for cryo-EM imaging of SOR are shown in **Table 1**. A representative micrograph in **Fig 1A** shows particles with a cage-like spherical structure and an inner central cavity. The micrographs were motion-corrected and the respective contrast transfer function (CTF) parameters were determined. The 2D classes showed internal structural details as depicted in **Fig 1B**. The particles from all the 2D classes in **Fig 1B** were 3D classified into three classes. The 3D classification generated a major class (Class 2) that exhibits the characteristic shape of SOR with six nanopores on its outer surface besides two other minor classes (1 & 3) showing incomplete particles (**Fig 1C**). Only the particles from Class 2 were used for further refinement. The resolution of the final cryo-EM map was 2.78 Å according to the estimation from the Fourier shell correlation (FSC) at the 0.143 cut-off criterion (**Fig 1D**). The color-coded local resolution cryo-EM map calculated by RELION is depicted in **Fig 1E**. The data processing pipeline flowchart for SOR is shown in **S2 Fig**.

**Table 1:** The table lists the microscope settings and the acquisition parameters of the cryo-EM micrographs for SOR and LS.

Hardware	SOR	LS
Microscope	Titan Krios	Titan Krios
Magnification	130,000	130,000
Voltage (kV)	300	300
Pixel size (Å)	1.06	1.06
Number of micrographs	800	1,516
**Data acquisition parameters**		
Dose per physical pixel per second (e/px.s)	4.76	3.92
Dose per Å^2^/sec	17	14
Exposure time (seconds)	4	5
Total dose (e/ Å^2^)	68	70
Number of fractions/frames	20	50
Dose per fraction (e/Å^2^)	3.4	1.4
**Latitude parameters**		
Defocus range (μm)	-0.5 to -1.5	-0.2 to -1.2
Defocus step (μm)	0.25	0.2
**Apertures size in μm**		
C1	2000	2000
C2	150	150
C3	2000	2000
Objective	100	100
Symmetry imposed	Octahedral	Icosahedral
Initial particle images (no.)	261,189	344,110
Final particle images (no.)	207,550	103,328
*B* factors (Å^2^)	-57.85	-59.31
Map resolution (Å)	2.78	2.33

**Fig 2:**
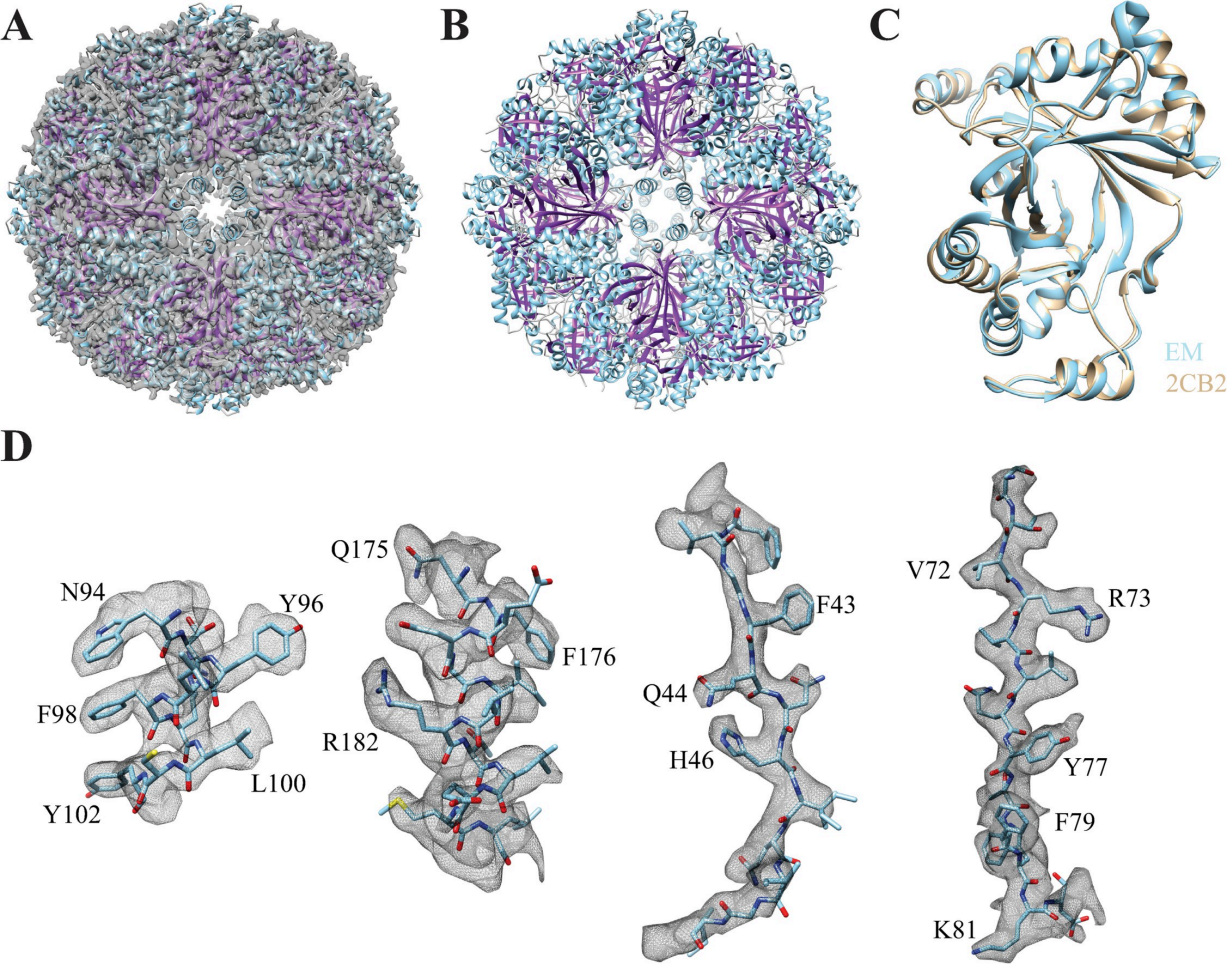
Structural details of SOR. (**A**) The atomic model (7X9W) of the 24 SOR monomers arranged in octahedral symmetry fitted inside the cryo-EM map. (**B**) Ribbon diagram of SOR atomic model. Each of the four identical subunits is viewed from the 4-fold axis. (**C**) The superposition of the atomic model of the monomer based on our cryo-EM map (cyan) onto the x-ray crystal structure (tan, 2CB2). The RMSD between the cryoEM and x-ray crystal structures is 0.575 Å for the 307 Cα atoms. (**D**) Representative secondary structural elements of SOR in stick representation fitted inside the respective regions of the cryo-EM map.

The monomeric subunits of SOR are arranged in the 432-point group symmetry forming a hollow sphere. The cryo-EM map fitted to the atomic model composed of 24 monomers arranged in octahedral symmetry is depicted in **Fig 2A**. The ribbon diagram of the atomic model viewed from the 4-fold symmetry axis shows four identical subunits (**Fig 2B**). The superposition of the monomeric subunit of the cryo-EM atomic model coincides with the x-ray crystal structure (2CB2) (**Fig 2C**). The average root mean square deviation (RMSD) between the cryoEM and x-ray crystal structures of SOR is 0.575 Å for the 307 Cα atoms. Both active site and side chains look very similar between the two structures. The reconstruction of the atomic model (7X9W) into the cryo-EM map and real space refinement were performed in PHENIX [60]. The model building and refinement statistics are shown in **Table 2**. Representative secondary structural elements fitted within the respective regions of the cryo-EM map are depicted in **Fig 2D**.

**Table 2:** Refinement statistics of the cryo-EM models.

	SOR	LS
	(EMDB-33084)	(EMDB-33041)
	(PDB 7X9W)	(PDB 7X7M)
**Initial PDB**	2CB2	1HQK
Model resolution (Å)		
FSC threshold 0.143/0.5	2.73/2.93	2.15/2.44
Map sharpening *B* factor (Å^2^)	15	60
**Model composition**		
Chains	24	60
Atoms	59640	70620
Protein residues	7368	9240
Ligands/Water	Fe (24) / HOH (48)	—
**R.m.s. deviations**		
Length (Å) (# > 4σ)	0.008	0.009
Angles (°) (# > 4σ)	1.189	0.698
**Validation**		
MolProbity score	2.26	1.28
Clash score	5.85	3.68
Rotamer outliers (%)	6.95	0.01
**Ramachandran plot**		
Favored/allowed/outlier (%)	96.21/3.79/0.00	97.37/2.63/0.00
**Model vs. Data**		
CC (mask)	0.83	0.90
CC (peaks)	0.75	0.85
CC (volume)	0.82	0.90

#### Structure of the catalytic site of SOR

**Fig 3:**
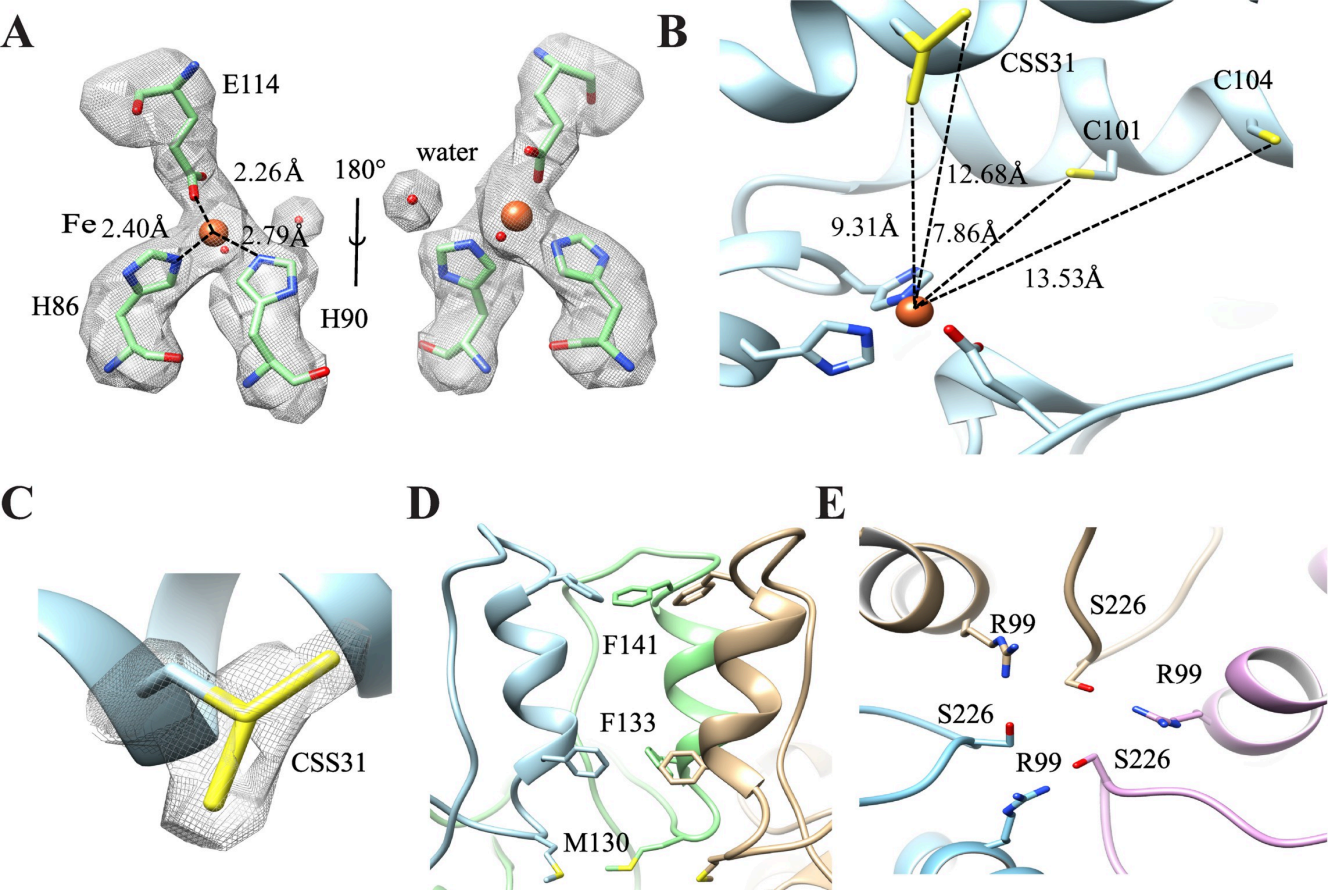
The catalytic pocket of SOR. (**A**) The mononuclear nonheme iron center in the active-site contains Fe^3+^ ion (orange sphere) ligated to the three conserved residues H86, H90 and E114. The distances between Fe^3+^ ion and the three nearby side chains are denoted. The catalytic site contains two water molecules (red spheres) that could be identified from the cryo-EM map as previously observed in the x-ray crystal structure [21]. A 180° view of the same region is shown. (**B**) Fe^3+^ is hexa-coordinated to three conserved cysteines and the H86, H90 and E114 residues. The distances between Fe^3+^ ion and the three conserved cysteines; pursulfurated cysteine 31 (CSS31), C101 and C104 are denoted. (**C**) The enlarged region shows extra density in the cryo-EM map due to the existence of CSS31 in two rotamer conformations with equal occupancies. (**D**) Side view of the channel formed at the 4-fold symmetry axis, where only three monomers are depicted for clarity. The outer, inner and bottom rings are formed by F141, F133, and M130, respectively. (**E**) Top view of the channel formed at the three-fold axis by R99 and S226 residues. The hydrophilic trimer channels are formed by three subunits positioned along a 3-fold symmetry axis and are proposed to be the exit routes of the polar products such as HSO_3−_, HS^−^ and S_2_O_3_^2−^.

In the oxygen-dependent sulfur disproportionation reaction, SOR catalyzes the oxidation and the reduction of elemental sulfur to produce sulfite, thiosulfate, and sulfide (**Eq 1**) [23, 28, 29]. The core structure of the monomer is composed of eight β-strands surrounded by nine α-helices [21]. The catalytic active site in each of the 24 monomeric subunits contains a mononuclear non-heme iron site [32], where the Fe^3+^ ion is responsible for the positive charge of the inner surface of the hollow cavity in SOR (**Fig 3A**). The density of the side chains of H86, H90 and E114 surrounding the iron center, and that of two water molecules ligated to the Fe^3+^ ion are distinguishable in our cryo-EM map (**Fig 3A**). The distances between Fe^3+^ ion and the three nearby side chains are denoted and a 180° view of the same region is shown. Biochemical studies revealed that mutations in the iron-coordinating amino acid residues H86, H90 and E114 result in iron loss and abolishment of the enzymatic activity [61]. Besides these three residues, there are three conserved cysteines; pursulfurated cysteine 31 (CSS31), C101 and C104 that are present in the active site and complete the hexa-coordination of the Fe^3+^ ion [61, 62] (**Fig 3B**). The mutation of C101 to S was associated with iron loss and resulted in near complete loss of activity [61]. In contrast, mutation of C101 to A resulted in higher residual activities and retention of approximately 50% of the iron content. The difference was attributed to the interference of the polar serine into iron incorporation compared to the apolar side chain of alanine [61]. The double mutations of both C101 and C104 residues did not completely abolish the activity, whereas mutation in CSS31 located on the entrance side of the monomer tunnel led to complete loss of enzymatic activity even though the iron content in this mutant was equivalent to that of wild-type SOR [61].


$$\begin{array}{ll} \text{Oxygenase} & \text{S}+{\text{O}}_{2}+{\text{H}}_{2}\text{O}\to {{\text{HSO}}_{3}}^{-}+{\text{H}}^{+}\\ \text{Disproportionation} & 3\text{S}+3{\text{H}}_{2}\text{O}\to 2{\text{HS}}^{-}+{{\text{HSO}}_{3}}^{-}+3{\text{H}}^{+}\\ \text{Sum} & 4\text{S}+{\text{O}}_{2}+4{\text{H}}_{2}\text{O}\to 2{\text{HS}}^{-}+2{{\text{HSO}}_{3}}^{-}+4{\text{H}}^{+}\\ \text{Non-enzymatic} & \text{S}+{{\text{HSO}}_{3}}^{-}\to {\text{S}}_{2}{{\text{O}}_{3}}^{2-}+{\text{H}}^{+} \end{array}$$ Eq 1

The extra density in the cryo-EM map (**Fig 3C**) results from CSS31 existing in two rotamer conformations with equal occupancies. It was proposed that the covalent binding of CSS31 to linear S substrates results in the formation of a polysulfide chain (R–S_*n*_–SH). Subsequently, the cysteine polysulfide undergoes hydrolytic cleavage to sulfide and a polysulfenyl moiety (R–S_*n*_–SOH), then the activation of oxygen occurs either by Fe^2+^ or the sulfenyl group [63]. However, this mechanism may not be universal since the conserved C31 is not persulfurated in SOR from *Acidianus tengchongensis*, which shares 88% sequence identity to SOR from *Acidianus ambivalens*. Besides, the iron atom in the active site of the former is penta-coordinated and ligated to one water molecule only [64].

#### Structure of the nano-channels

The characteristic feature for SOR is the presence of chimney-like protrusions at the 4-fold symmetry axes. These tetramer channel structures formed by the side chain of residues at each of four adjacent subunits are proposed to allow the sulfur substrates to access the inside of the hollow sphere cavity of SOR, where catalysis can take place secluded from the cytoplasmic environment [64]. The inner surface of the channel is hydrophobic to suit sulfur entry where the outer, inner and bottom rings are formed by the F141, F133 and M130 residues, respectively, as shown in **Fig 3D**. The mutation of the individual phenylalanine residues F133 and F141 into alanine resulted in less than 2-fold increase in specific enzyme activities. The double mutant exhibited higher catalytic activities (194% of the oxygenase and 347% of the reductase) [31]. Substrate for SOR should be linear polysulfide because of the limited size of the pores formed by phenylalanine rings [31]. The deletion of all or part of the amino acid residues that form the protrusions including both phenylalanine rings resulted in 7-fold increase in the enzymatic activities indicating that the substrate access to the active site is controlled by the tetramer channel [31, 65].

The exit routes of the polar products such as HSO_3−_, HS^−^ and S_2_O_3_^2−^ are proposed to be the hydrophilic trimer channels formed by three subunits positioned along a 3-fold symmetry axis [31]. The small channel formed by R99 and S226 residues of the subunit interface of the trimer channel is shown in **Fig 3E**. The amino acids R99 and S226 constitute the central elements of the subunit interface of the trimer channel at the three-fold symmetry axis [65]. Except for R99, the other residues in the trimer channel are not conserved. Mutations in R99 and S226 showed elevated enzyme activities, where R99A and S226T had ~ 182% oxygenase and 156% reductase activities [31].

### Cryo-EM structure of Lumazine Synthase (LS)

**Fig 4:**
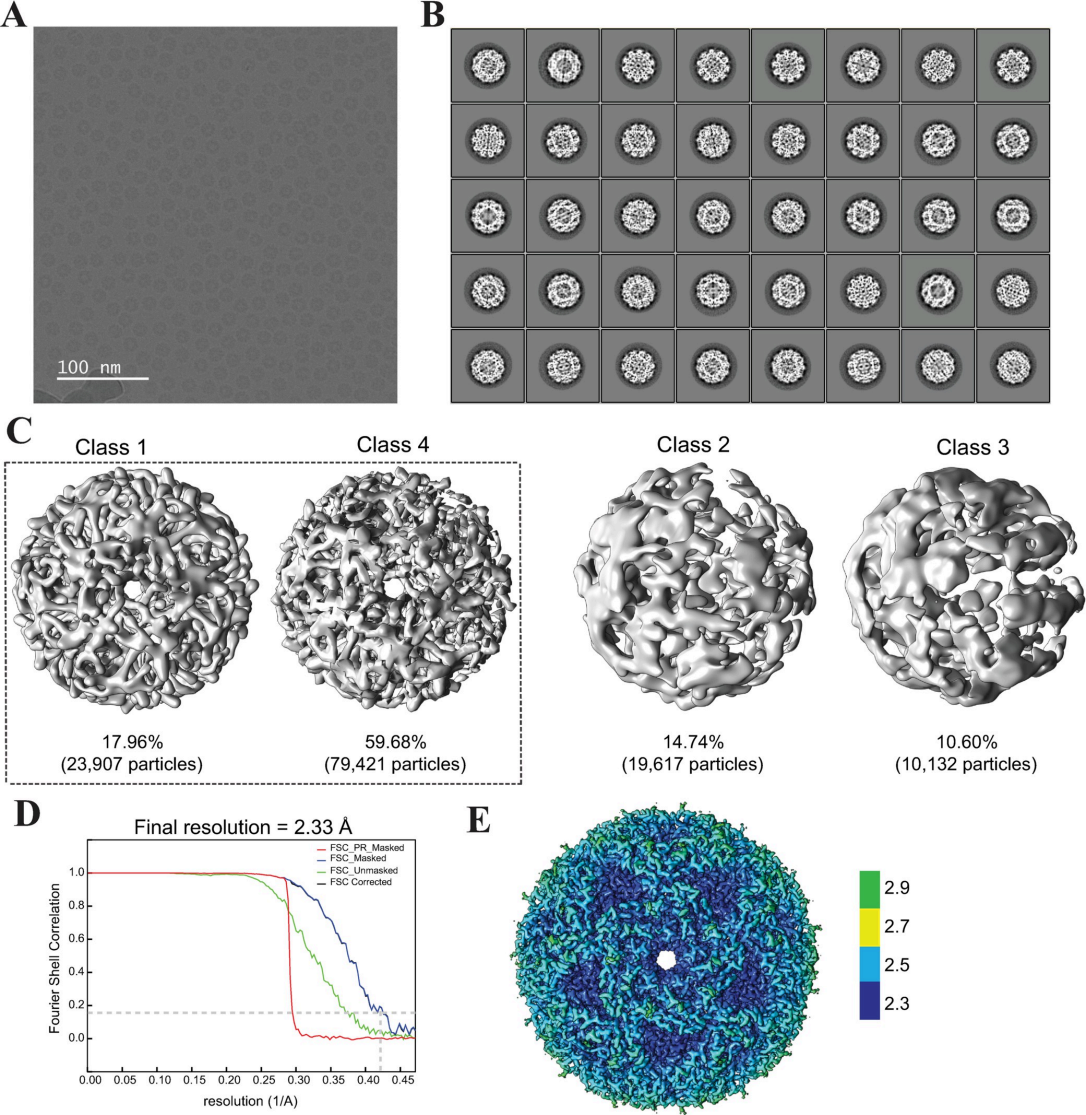
Cryo-EM imaging and 3D reconstruction of Lumazine Synthase (LS) cryo-EM map. (**A**) Representative micrograph of LS enzyme acquired in super-resolution mode and at 130 kx magnification. The scale bar represents 100 nm. (**B**) The 2D classes selected for 3D classification contained 133,077 particles distributed at random orientations. (**C**) The selected 2D classes were 3D classified into four classes. Classes 1 and 4 represented 77.64% of the total number of particles and showed the characteristic channel parallel to the 5-fold pentamer axis. The particles from both classes 1 and 4 were used for subsequent refinement. (**D**) The FSC plot shows the resolution of unmasked and masked maps after post-processing in RELION. The final resolution of the cryo-EM map is 2.33 Å at FSC = 0.143. (**E**) The cryo-EM map is color-coded with the local resolution estimate obtained from RELION. The data processing pipeline flowchart for LS is depicted in **S4 Fig**.

The cryo-EM micrographs of LS were acquired as described in the Materials and Methods section using the settings reported in **Table 1**. The size-exclusion chromatogram and SDS-PAGE gel image for the LS sample before grid freezing are shown in **S3 Fig**. The LS complex appeared in the micrographs as spherical particles with hollow centers (**Fig 4A**). **Fig 4B** shows the 2D classes selected for 3D classification. The 2D classes exhibited random orientations with high detail of the internal structure of LS. The 3D classification generated four classes; where Classes 1 and 4 showed similar resolution and displayed the characteristic channel parallel to the 5-fold pentamer axis (**Fig 4C**). We did not find any obvious different conformations in the 3D classes. The bad particles were excluded and only Classes 1 and 4 were used for subsequent refinement. Further processing and refinement resulted in a cryo-EM map at a global 2.33 Å resolution as depicted in the FSC plot (**Fig 4D**). The final cryo-EM map color-coded with local resolution as obtained in RELION is shown in **Fig 4E.** The data processing pipeline flowchart for LS is depicted in **S4 Fig**.

**Fig 5:**
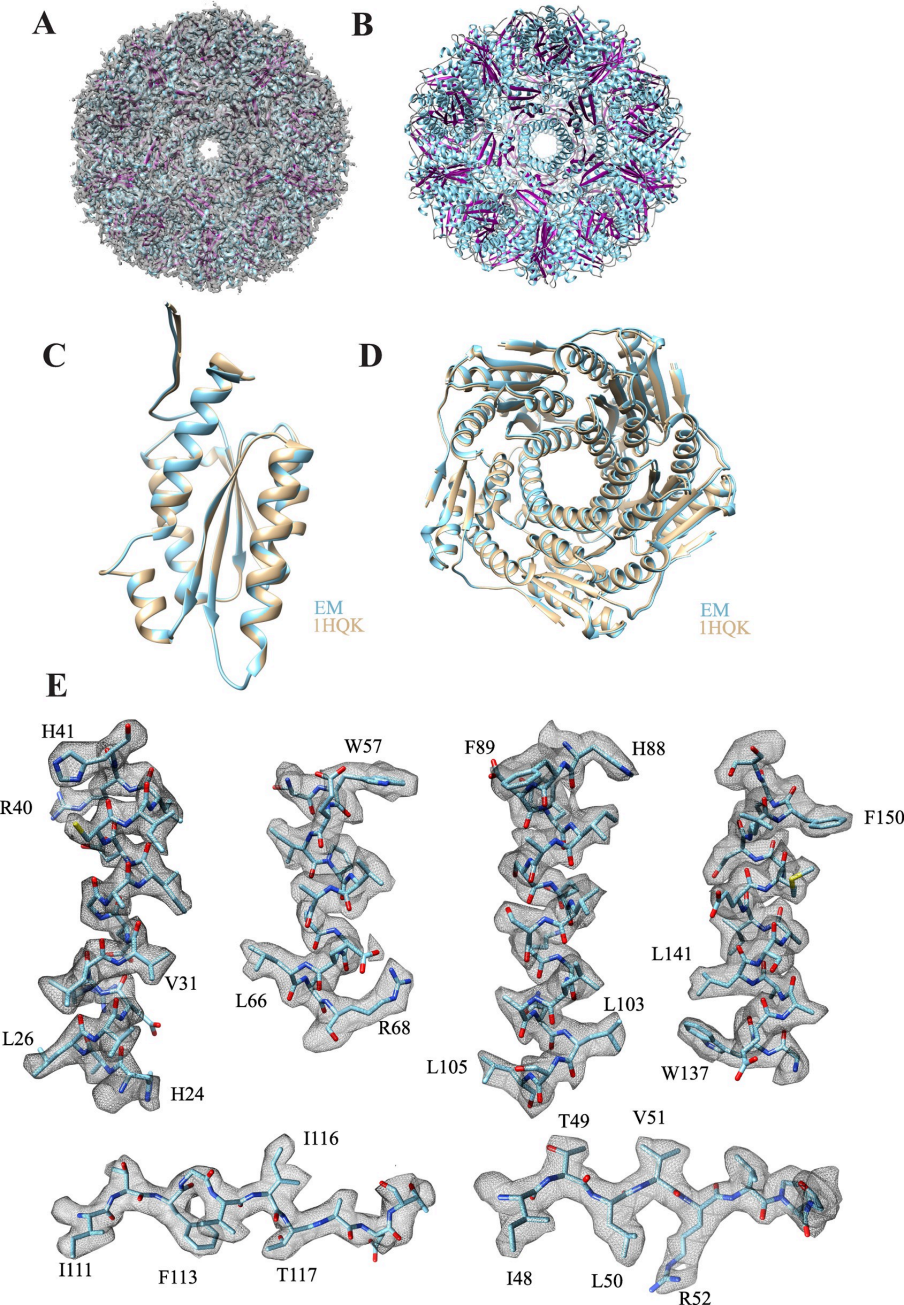
Structural details of LS. (**A**) The atomic model (7X7M) composed of 60 monomers arranged in icosahedral symmetry is superimposed into the cryo-EM map. (**B**) The ribbon diagram of LS is viewed from the 5-fold axis. (**C**) The atomic model of the monomer based on the cryo-EM map (cyan) is superimposed on the x-ray crystal structure (tan, 1HQK). The RMSD of both structures is 0.174 Å for the 154 Cα atoms. (**D**) The pentamer of LS based on the cryo-EM map (cyan) coincides with that of the x-ray crystal structure of *Aquifex aeolicus* LS (tan, 1HQK). **(E)** Representative secondary structural elements of the LS complex are shown within the cryo-EM map.

LS consists of 60 identical subunits that form a capsid of icosahedral 532 symmetry with an outer diameter of 154 Å and inner compartment diameter of 80 Å [44]. **Fig 5A** depicts the cryo-EM density map superimposed on the atomic model constituted of 60 monomeric subunits arranged in icosahedral symmetry. The ribbon diagram viewed from the 5-fold axis is shown in **Fig 5B**. The LS monomer is composed of a central four-stranded β-sheet flanked on one side by two α helices and on the other side by three α helices [40]. The monomeric subunit of the atomic model obtained from the cryo-EM map is superimposed on the x-ray crystal structure (1HQK) (**Fig 5C**). The RMSD of both structures is 0.174 Å for the 154 Cα atoms. The active site and side chains coincide between the two structures. Previous studies showed that a pentamer formed of LS monomers is the primary building block of the icosahedral complex. The LS pentamers from the cryo-EM and x-ray crystal structures are superimposable (**Fig 5D**). There is a channel in the center of the pentamer formed by an α helix (α3) and its four symmetry-related neighbors, with a diameter of about 9 Å, where most of the amino acids inside the channel are hydrophilic. Representative secondary structural elements of LS complex within the cryo-EM map are shown in **Fig 5E**.

#### Subunit interface, ion-pairs and ion-pair networks

**Fig 6:**
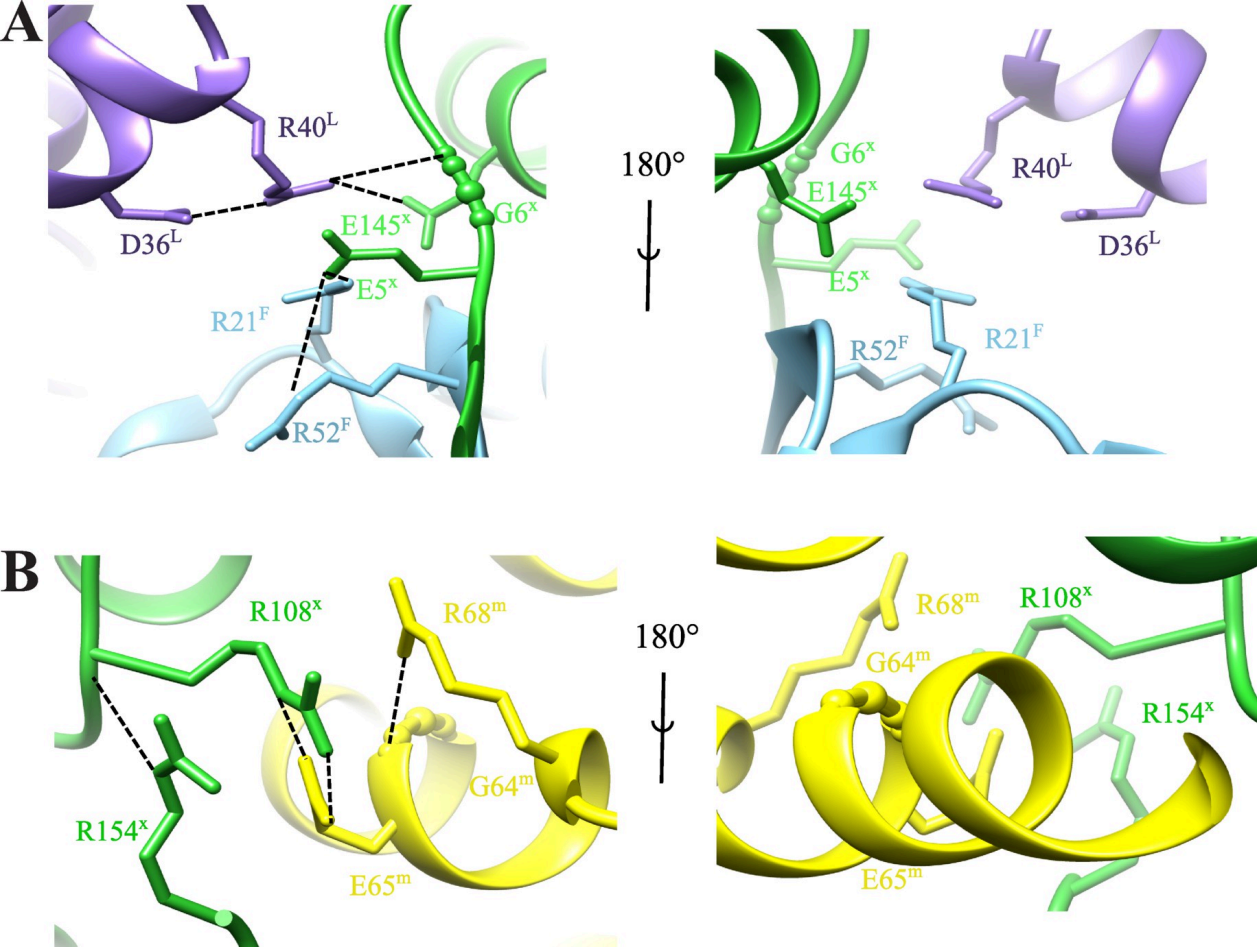
The ion-pair network structure in LS. (**A**) The inter-chain interactions at the interface between two pentamers involve six residues from three LS monomers. The inter-chain interactions between the residues in chain L (purple) from the first pentamer and chains F (green) and x (cyan) from the second pentamer are illustrated. The chain identifier is added to each residue in superscript. The residue R40^L^ forms salt-bridges with residues D36^L^, E145^x^ and a hydrogen bridge with residue G6^x^. The residue R21^F^ forms an ion-pair with E5^x^ which itself interacts with R52^F^. A 180° rotated view of the same region is shown. (**B**) The intra-chain interactions between two LS monomers within the same pentamer are built up by the three arginine residues R68, R108 and R154. The R108^x^ residue interacts with R154^x^ within the same chain and forms an ionic contact with E65^m^ from another chain. The R68^m^ residue forms a main-chain contact to the oxygen in G64^m^. A 180° view of the interface between the two LS monomers is shown.

Structural comparison of proteins from mesophiles and hyperthermophiles has illustrated that the thermal stability of proteins correlates with the number of surface ion-pairs [66]. Compared to other lumazine synthases, LS from *Aquifex aeolicus* has the largest accessible surface formed by charged residues, the smallest surface formed by hydrophobic residues, and the largest number of ion-pairs per subunit [46]. Therefore, the exceptional thermostability of LS from *Aquifex aeolicus* was attributed to the optimal hydrophobic and ionic contacts [44]. The inter-chain interactions at the interface between two pentamers are stabilized by six residues connecting three LS monomers as shown in **Fig 6A**. The inter-chain interactions between the residues in chain L (purple) from the first pentamer and chains F (green) and x (cyan) from the second pentamer are illustrated. The chain identifier is added to each residue in superscript. The residue R40^L^ forms salt-bridges with residues D36^L^, E145^x^ and a hydrogen bridge with residue G6^x^, while the residue R21^F^ forms an ion-pair with E5^x^ which itself interacts with R52^F^. A 180° rotated view of the same region is shown (**Fig 6A**). The intra-chain interactions between two LS monomers within the same pentamer are depicted in **Fig 6B**. These interactions are built up by the three arginine residues R68, R108 and R154. The R108^x^ residue interacts with R154^x^ within the same chain and forms an ionic contact with E65^m^ from another chain, while R68^m^ forms a main-chain contact to the oxygen in G64^m^ [44]. A 180° view of the interface between the two LS monomers is shown in **Fig 6B**. These different types of inter- and intra-chain interactions may explain the exceptional thermostability of the LS from *Aquifex aeolicus*.

## Discussion

Enzymes are biocatalysts that perform all the chemical and biological reactions including metabolism and energy production in living organisms. The intricate structures of enzymes are specifically tailored to suit their functions. Enzymes drive the chemical reactions at a much lower energy barrier and under milder conditions, thus alleviating the need for harsh conditions such as elevated temperatures or highly acidic or alkaline conditions inside the cells. In many biotechnological fields, there have been ongoing efforts to harness the power of enzymes in driving demanding reactions at much milder conditions and fewer steps thus lowering the total expense. Given the wide variations in the environments of living organisms, many extremophilic microorganisms have been identified and found to contain enzymes capable of functioning under extreme conditions such as extreme temperatures and salinity. The understanding of the way these enzymes can function under such conditions requires thorough investigation of their structures and characteristics. Cryo-EM is becoming increasingly important for studying proteins and protein complexes. Unlike x-ray crystallography which requires the crystallization as a prerequisite for structural determination that may not be possible in some macromolecular complexes, cryo-EM does not have this condition and enables the study of proteins in their native aqueous environment.

In this study, we presented the cryo-EM structures of two thermostable multimeric enzymes; namely SOR from *Acidianus ambivalens* (7X9W) and LS from *Aquifex aeolicus* (7X7M) at 2.78 Å and 2.33 Å resolution, respectively. The 3D classification did not show any obvious different conformations and the two samples were homogeneous. Overall, we could not find significant differences between our cryo-EM structures and those determined by x-ray crystallography. Therefore, the high resolution x-ray crystal structures provided validation for our cryo-EM structures. At present, cryo-EM is becoming more accessible to a wide group of users from different fields, therefore the field of thermostable enzymes can benefit from the advancements in cryo-EM. In exchange, the extreme thermostability, optimum size and high symmetry of these enzymes render them suitable candidates for benchmarking cryo-EM through obtaining better cryo-EM maps and high resolution structures. Besides, thermostable enzymes are very stable samples that can be easily purified. Therefore, we hope this study provides motivation for the readers to explore other thermostable proteins using cryo-EM.

## Materials and methods

### Protein expression and purification

The amino acid sequence of Sulfur Oxygenase Reductase gene (Uniprot P29082) of *Acidianus ambivalens* was codon optimized for expression in *Escherichia coli*. The optimized coding sequence was fused to Streptag at the C-terminus of the protein. The gene was synthesized by Integrated DNA Technologies (IDT) and cloned into a vector by Gibson assembly reaction. The plasmid was transformed into OneShot competent cells. Several colonies were randomly selected and the sequence of the extracted plasmids was verified by Sanger sequencing. Four 2L cultures of transformed BL21 (DE3) cells in LB media were grown at 37°C until the culture reached an optical density at 600 nm wavelength (OD_600_) of 0.8. Protein expression was induced by adding 0.2 mM IPTG at 16°C for the duration of 17 hours. The cells were spun down at 5,000 x g for 30 minutes. The cell pellet was resuspended in lysis buffer (100 mM potassium phosphate (KP_i_) buffer pH 8, 1% (v/v) Tween 20 and 50 μM EDTA) at 4 mL per gm of cells. One protease inhibitor cocktail tablet (ThermoFisher Scientific) was added to each 50 mL of cell suspension. The cells were disrupted by ultrasonication at 10 sec on, 20 sec off for 2 minutes at 35% power repeated three times, then spun down at 22,040 xg for 30 minutes. The supernatant was heated at 75°C for 45 min then spun down at 35,000 rpm in 45 Ti rotor in Beckman Coulter centrifuge for 1 hour at 4°C then filtered through 45 μm filter to remove precipitated heat-labile proteins. The filtrate was passed through StrepTrap HP 5 mL column (GE Healthcare) using 100 mM Tris-HCl pH 8.0 and 150 mM NaCl (Buffer A). SOR was eluted by Buffer B (Buffer A + 2.5 mM d-desthiobiotin). The fractions containing the protein were collected and concentrated to 700 μL using 100 kDa cutoff Amicon concentrator and loaded onto Superdex 200 10/300 GL column (GE Healthcare) using 20 mM Tris-HCl pH 7.5 and 150 mM NaCl. The fractions containing the eluted protein were collected and concentrated using 100 kDa cutoff Amicon concentrator then flash frozen into liquid nitrogen and stored at -80°C. The size-exclusion chromatogram and SDS-PAGE gel image for the SOR sample before grid freezing are shown in **S1 Fig**.

The amino-acid sequence of the Lumazine Synthase gene *from Aquifex aeolicus* (Uniprot O66529) with Streptag fused to the C-terminal was codon-optimized for expression in *Escherichia coli*. The gene was ordered from IDT. The cloning of the LS gene, plasmid transformation, and protein expression and purification were performed similarly to SOR. The size-exclusion chromatogram and SDS-PAGE gel image for the LS sample before grid freezing are shown in **S3 Fig**.

### Cryo-EM imaging and model reconstruction

Quantifoil R2/2 300-mesh gold grids were glow-discharged at 30 mA for 1 minute. The chamber of the Vitrobot Mark II (ThermoFisher Scientific) was kept at 22°C and 100% humidity. A volume of 2 μL of the purified protein at 6 mg/mL concentration was applied to the glow discharged grids inside the chamber, blotted for 1.5 s, and then plunge-frozen in liquid ethane cooled by liquid nitrogen. The grids were then clipped and loaded into a Titan Krios microscope (ThermoFisher Scientific) operating at 300 kV. The data sets were acquired at a nominal magnification of 130,000x (0.53 Å/pixel) using a K2 Summit direct electron detector camera (Gatan, Inc.) operating in super-resolution mode. Data were collected using LatitudeS software (Gatan, Inc.), using the defocus range shown in **Table 1**. The total dose was fractionated over multiple frames during the exposure time. Data processing was performed using RELION-3.0 [53] installed on a dedicated central CPU and GPU cluster. The whole-frame alignment of the dose-fractionated movies and 2-binning were carried out using MotionCor 2.1 [67]. The motion-corrected micrographs were then used to determine the contrast transfer function (CTF) using GCTF v1.18 [68]. The data processing pipeline flowchart for SOR is depicted in **S2 Fig**. In the case of SOR, the reference-free auto-picking procedure based on a Laplacian-of-Gaussian filter (LoG) picked 391,093 particles from 800 micrographs. Two consecutive rounds of 2D classifications were performed on the selected particles. The selected highest quality 2D classes showed detailed internal structure and contained 261,189 particles. The 3D initial model was generated from the selected 2D particles using Stochastic Gradient Descent (SGD) algorithm in RELION. The data was processed without symmetry (C1 symmetry) until the 3D classification then the octahedral symmetry parameter (O) was applied during the 3D refinement of SOR. The 3D classification into 3 classes resulted in 79.46% of particles in Class 2 (207,550), 15.26% of particles in Class 1 (39,864), and 5.27% of particles in Class 3 (13,775). Class 2 was selected for further 3D refinement. The per-particle defocus values and beam tilt values for the data set were estimated by CTF refinement. The Bayesian polishing (a Bayesian approach for beam-induced motion correction) as implemented in RELION was run in training mode on a subset of 10,000 particles. The output parameters were then used by the program to fit tracks for the motion of all particles in the data set and to produce adequately weighted averages of the aligned movie frames followed by 3D refinement of the polished particles. The mask was generated using a 15 Å low-pass filter, the binary map was extended by 4 pixels and a soft-edge of 6 pixels was applied. The map was sharpened using the modulation transfer function (MTF) curve of Gatan K2 summit camera and the global β-factor correction based on Guinier fitting implemented in the standard post-processing procedure of RELION. The same mask used during the initial 3D refinement was applied during post-processing. The FSC curves were calculated in RELION. The final sharpened/post-processed map at 2.78 Å resolution was deposited in the Electron Microscopy Data Bank (EMDB) repository.

The processing of the data set for LS was performed similar to SOR. The data processing pipeline flowchart for SOR is depicted in **S4 Fig**. Briefly, in the case of LS, 344,110 particles were selected by LoG picking from 1,516 micrographs. After 2D classification 133,077 particles were selected and used for 3D classification using 4 classes. The combined particles from Class 1 and Class 4 (103,328) constituted 77.64% of the total number of particles. The data was processed with C1 symmetry until the 3D classification then icosahedral (I4) symmetry was applied during 3D refinement. The mask was generated using the aforementioned parameters used in the case of SOR. CTF refinement and Bayesian polishing were performed on the data set followed by 3D refinement of the polished particles. The resolution of sharpened, masked and post-processed map was estimated at 2.33 Å using the gold-standard FSC method.

### Map refinement and model building

The model reconstruction into the cryo-EM map and real space refinement were performed in PHENIX [60] using the starting atomic models 2CB2 and 1HQK for SOR and LS, respectively. Structural validation was also performed in PHENIX using molprobity-based “Comprehensive validation” [69]. Maps and models were visualized using UCSF Chimera [70], ChimeraX [71] and Coot [72]. The refinement statistics of the cryo-EM models 7X9W and 7X7M for SOR and LS, respectively, are listed in **Table 2**.

## Supporting information

S1 Fig The purification of Sulfur oxygenase reductase (SOR). (A) The size-exclusion chromatogram of SOR using Superdex 200 10/300 GL column (GE Healthcare). (B) SDS-PAGE of the respective fractions showing the intense band of SOR subunit at the molecular weight of ~ 35 kDa.
(TIF)

S2 Fig The data processing pipeline for the cryo-EM movies of Sulfur oxygenase reductase (SOR). The flow chart shows the image-processing steps starting from the acquired micrographs till the final cryo-EM map of SOR at 2.78 Å resolution.
(TIF)

S3 Fig The purification of Lumazine synthase (LS). (A) The size-exclusion chromatogram of LS using Superdex 200 10/300 GL column (GE Healthcare). (B) SDS-PAGE of the respective fractions showing the intense band of the LS monomer at the molecular weight of ~ 16 kDa.
(TIF)

S4 Fig The data processing pipeline for the cryo-EM movies of Lumazine synthase (LS). The flow chart shows the image-processing steps starting from the acquired micrographs till the final cryo-EM map of LS at 2.33 **Å** resolution.
(TIF)
