# Supplementary figures and images for: Cryo-electron structures of the extreme thermostable enzymes Sulfur Oxygenase Reductase and Lumazine Synthase
Source: PLoS One. 2022 Oct 3;17(10):e0275487. doi: 10.1371/journal.pone.0275487 (PMC9529111; doi:10.1371/journal.pone.0275487)

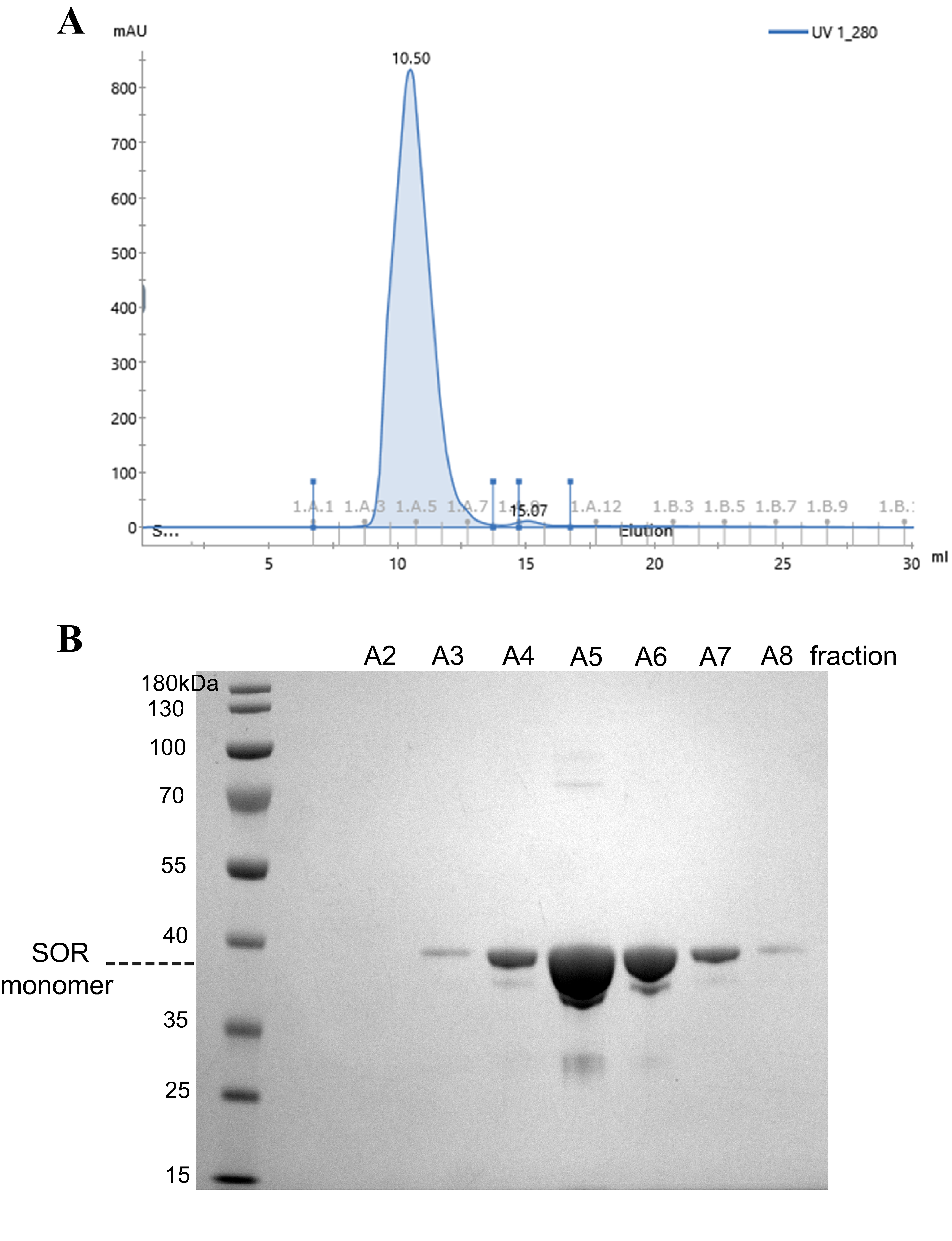

Supplement: S1 Fig — (A) The size-exclusion chromatogram of SOR using Superdex 200 10/300 GL column (GE Healthcare). (B) SDS-PAGE of the respective fractions showing the intense band of SOR subunit at the molecular weight of ~ 35 kDa. (TIF) [file pone.0275487.s001.tif]

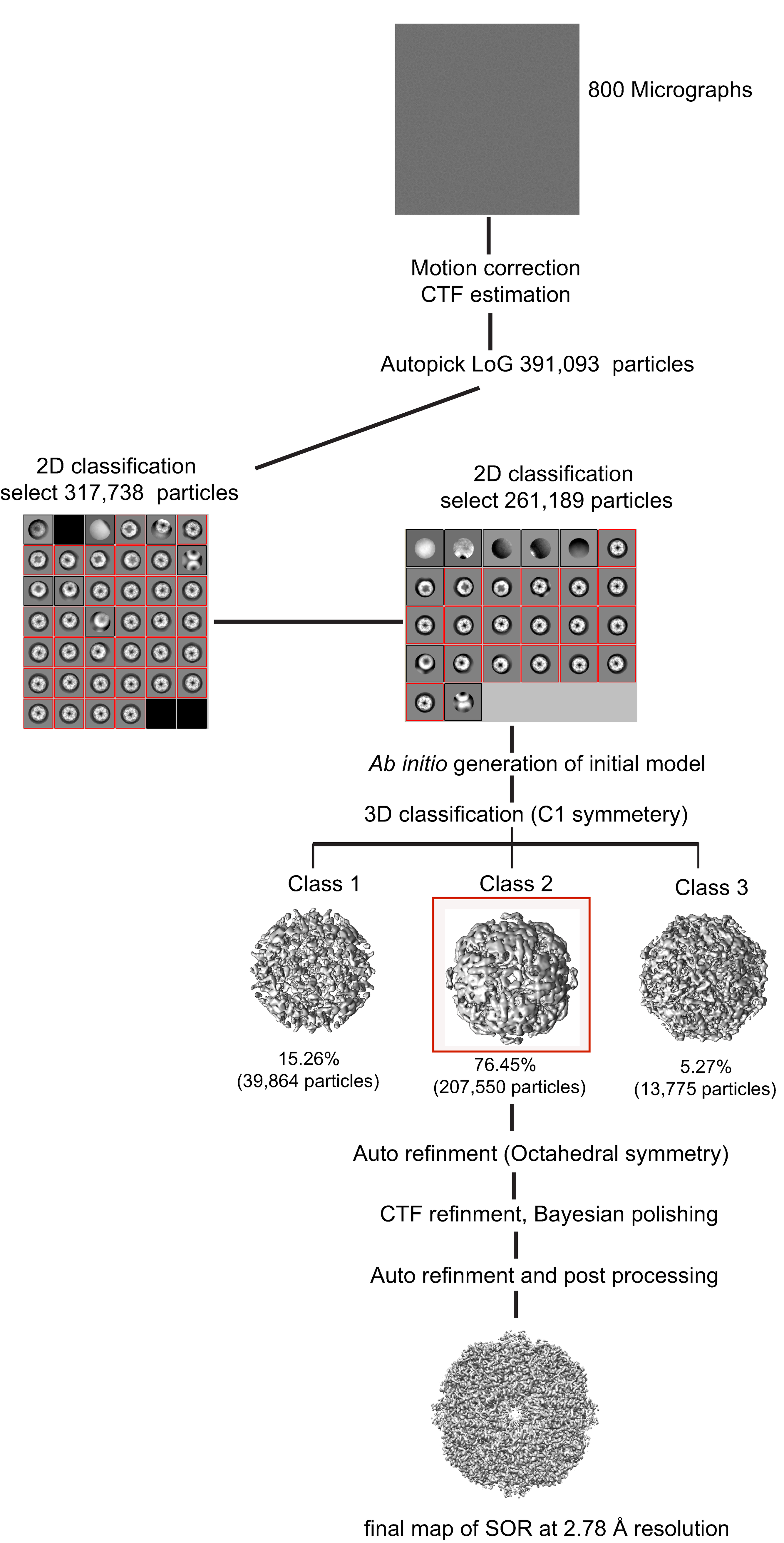

Supplement: S2 Fig — The flow chart shows the image-processing steps starting from the acquired micrographs till the final cryo-EM map of SOR at 2.78 Å resolution. (TIF) [file pone.0275487.s002.tif]

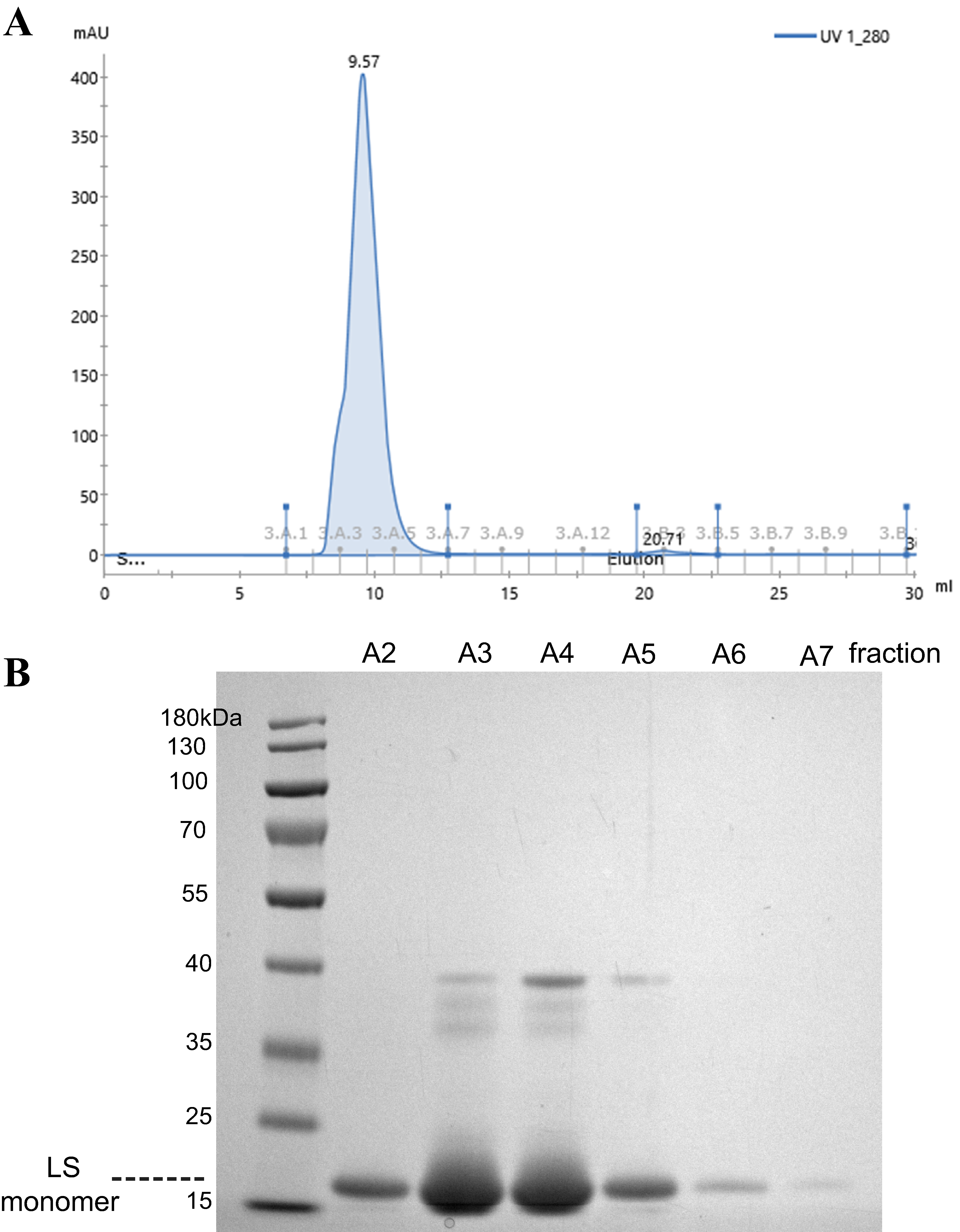

Supplement: S3 Fig — (A) The size-exclusion chromatogram of LS using Superdex 200 10/300 GL column (GE Healthcare). (B) SDS-PAGE of the respective fractions showing the intense band of the LS monomer at the molecular weight of ~ 16 kDa. (TIF) [file pone.0275487.s003.tif]

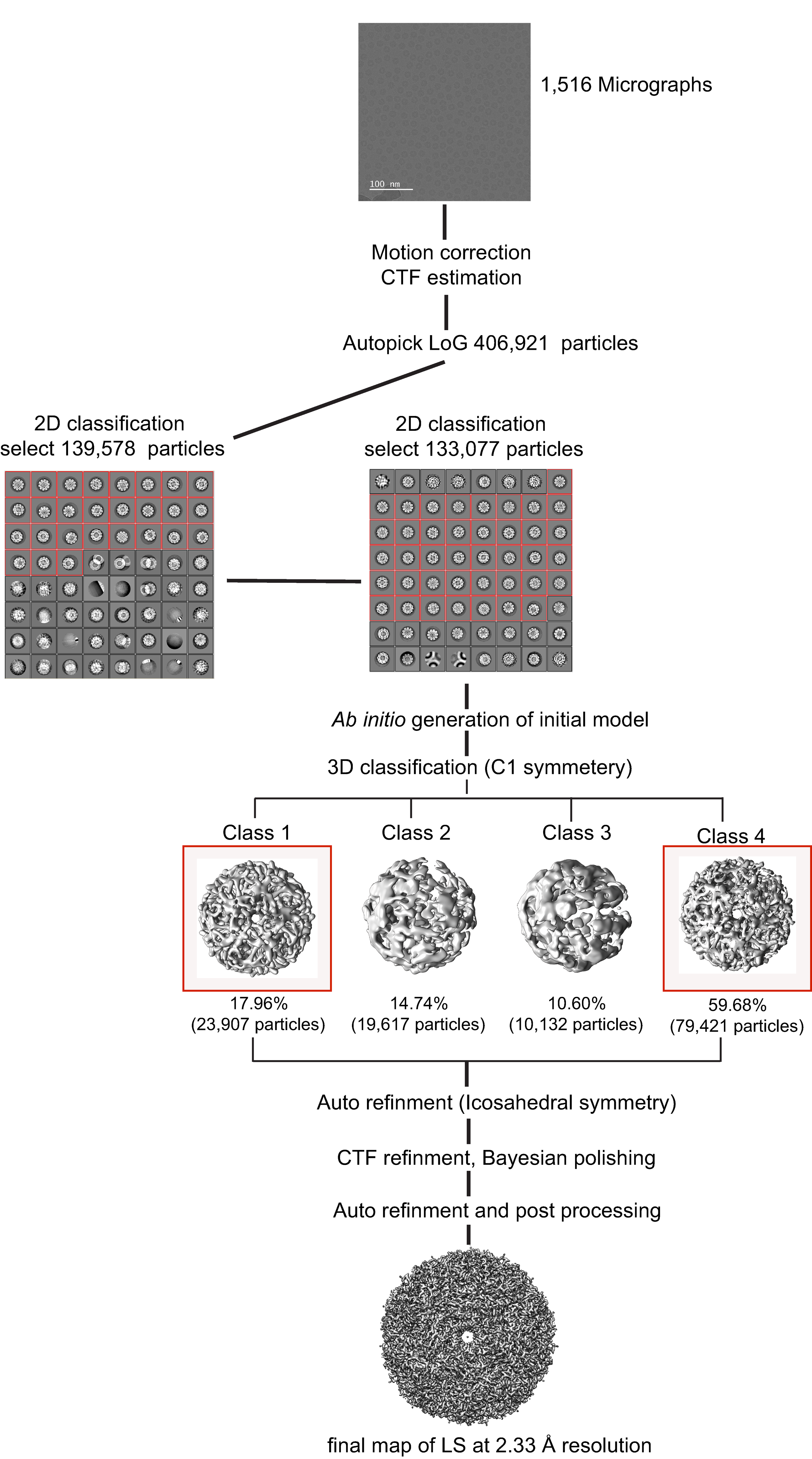

Supplement: S4 Fig — The flow chart shows the image-processing steps starting from the acquired micrographs till the final cryo-EM map of LS at 2.33 Å resolution. (TIF) [file pone.0275487.s004.tif]
